# Bone Marrow Pathology Predicts Mortality in Chronic Hemodialysis Patients

**DOI:** 10.1155/2015/160382

**Published:** 2015-02-24

**Authors:** Cheng-Hao Weng, Kuan-Ying Lu, Ching-Chih Hu, Wen-Hung Huang, I-Kwan Wang, Tzung-Hai Yen

**Affiliations:** ^1^Department of Nephrology and Division of Clinical Toxicology, Chang Gung Memorial Hospital, Linkou 333, Taiwan; ^2^College of Medicine, Chang Gung University, Taoyuan 333, Taiwan; ^3^Kidney Research Center, Chang Gung Memorial Hospital, Linkou 333, Taiwan; ^4^Department of Hepatogastroenterology and Liver Research Unit, Chang Gung Memorial Hospital, Keelung 204, Taiwan; ^5^Department of Nephrology, China Medical University Hospital, Taichung 404, Taiwan; ^6^College of Medicine, China Medical University, Taichung 404, Taiwan; ^7^Center for Tissue Engineering, Chang Gung Memorial Hospital, Linkou 333, Taiwan

## Abstract

*Introduction*. A bone marrow biopsy is a useful procedure for the diagnosis and staging of various hematologic and systemic diseases. The objective of this study was to investigate whether the findings of bone marrow studies can predict mortality in chronic hemodialysis patients. *Methods*. Seventy-eight end-stage renal disease patients on maintenance hemodialysis underwent bone marrow biopsies between 2000 and 2011, with the most common indication being unexplained anemia followed by unexplained leukocytosis and leukopenia. *Results*. The survivors had a higher incidence of abnormal megakaryocyte distribution (*P* = 0.001), band and segmented cells (*P* = 0.021), and lymphoid cells (*P* = 0.029) than the nonsurvivors. The overall mortality rate was 38.5% (30/78), and the most common cause of mortality was sepsis (83.3%) followed by respiratory failure (10%). In multivariate Cox regression analysis, both decreased (OR 3.714, 95% CI 1.671–8.253, *P* = 0.001) and absent (OR 9.751, 95% CI 2.030–45.115, *P* = 0.004) megakaryocyte distribution (normal megakaryocyte distribution as the reference group), as well as myeloid/erythroid ratio (OR 1.054, CI 1.012–1.098, *P* = 0.011), were predictive of mortality. *Conclusion*. The results of a bone marrow biopsy can be used to assess the pathology, and, in addition, myeloid/erythroid ratio and abnormal megakaryocyte distribution can predict mortality in chronic hemodialysis patients.

## 1. Introduction

A bone marrow biopsy is a useful procedure for the diagnosis and staging of various hematologic diseases and in the assessment of bone marrow cellularity, cellular morphology, maturation, and the possibility of occult infection [1]. In ESRD patients, bone marrow studies are especially useful to evaluate the iron store in patients with anemia. Anemia in ESRD patients results from the reduced kidney production of erythropoietin (EPO) and changes in iron homeostasis which can lead to iron deficiency. Thus, routine monitoring of iron store is crucial for the adequate management of anemia in these patients [2]. Bone marrow fibrosis with a concomitant reduction in space for erythrogenesis induced by secondary hyperparathyroidism increases the dose of EPO required to maintain an adequate response [3]. The finding of bone marrow fibrosis in bone marrow studies in ESRD patients with anemia can allow for the administration of an adequate dosage of calcitriol or its analogs or parathyroidectomy. Except for those with anemia, other indications for bone marrow studies in ESRD patients are the same as in the general population. Therefore, the objective of this study was to investigate whether the findings of bone marrow studies can predict mortality in chronic hemodialysis patients.

## 2. Material and Methods

### 2.1. Ethics Statement

This retrospective observational study complied with the guidelines of the Declaration of Helsinki and was approved by the Medical Ethics Committee of Chang Gung Memorial Hospital, a tertiary referral center located in northern Taiwan. Since this study involved a retrospective review of existing data, approval from the Institutional Review Board was obtained, but without specific informed consent from the patients. Furthermore, all data were securely protected (by delinking identifying information from the main data sets) and made available only to investigators, and they were also analyzed anonymously. The Institutional Review Board of Chang Gung Memorial Hospital specifically waived the need for consent for these studies. Finally, all primary data were collected according to procedures outlined in epidemiology guidelines that strengthen the reporting of observational studies. This policy was based on previous publications [4–6].

### 2.2. Patients

In total, 78 ESRD patients on maintenance hemodialysis underwent bone marrow biopsies between 2000 and 2011. Demographic, hematological, biochemical, and dialysis related data were obtained at the time of the bone marrow biopsies for cross-sectional analysis. Causes of death and mortality rates were also analyzed for each subgroup.

### 2.3. Inclusion and Exclusion Criteria

Patients were included in this study if they were more than 18 years of age and if they had undergone hemodialysis for at least 3 months. Patients with the final diagnosis of hematological diseases such as leukemia and multiple myeloma were excluded. The indications for the bone marrow biopsy are listed in Table 2.

### 2.4. Bone Marrow Biopsy Specimen Preparation

The procedure was based on the guidelines published by the International Council for Standardization in Hematology [7]. Bone marrow biopsy specimens were processed with fixation and decalcification. After decalcification, the specimens were embedded in paraffin wax and sections were cut on a microtome. The biopsy sections were stained with hematoxylin and eosin.

### 2.5. Bone Marrow Biopsy Sections Microscopy Examination

The procedure was based on the guidelines published by the International Council for Standardization in Hematology [7]. Two to four sections were routinely reviewed. The percentage of cellularity was obtained by estimating the proportion of cells occupying the total marrow cavity. The sections were viewed initially at low power (×40–×100) for adequacy, pattern, cellularity, presence of focal lesions, number of megakaryocytes, abnormal cell clusters and location, bone structure (trabecular number and thickness), and osteoclastic and osteoblastic activity. The sections were subsequently viewed under higher magnification (×200–×400) to assess hematopoietic activity (e.g., erythroid, myeloid, megakaryocytic lineages, lymphoid cells, plasma cells, and macrophages) and cytological detail. Higher magnifications of ×600–×1000 were used to assess fine cytological details such as intracellular granules and Auer rods.

### 2.6. Definition of Bone Marrow Cellularity

Bone marrow contains hematopoietic stem cells and stromal cells (mostly adipocytes) [8], and marrow cellularity is the volume ratio of hematopoiesis and fat. The normal cellularity of adult hematopoietic bone marrow ranges from 30 to 70%, and this changes under pathological conditions. Hypercellular marrow is defined as more than 70%, normocellular marrow as 30–70%, and hypocellular marrow as under 30% bone marrow [9].

### 2.7. Definition of Normal, Increased, and Decreased Megakaryocyte Distribution

Normally, about 5 to 10 megakaryocytes are seen per microscopic field at low power magnification (10x objective). Clusters of megakaryocytes usually indicate megakaryocytic hyperplasia or increased megakaryocyte distribution. Less than 2 megakaryocytes per low power field means megakaryocytic hypoplasia [9] or decreased megakaryocyte distribution. An abnormal megakaryocyte distribution was defined as an increase, decrease, or absence of the distribution of megakaryocytes.

### 2.8. Definition of Mortality and Survival

The definition of mortality and survival in this study was mortality or survival after the bone marrow biopsy procedure.

### 2.9. Definition of Hemodialysis Adequacy *Kt*/*V*

The *Kt*/*V* is used to quantify the adequacy of hemodialysis treatment, where *K* represents the dialyzer clearance of urea, *t* represents dialysis time, *V* represents the volume of distribution of urea which is approximately equal to the patient's total volume of body water [10].

### 2.10. Statistical Analysis

Data were expressed as mean ± standard deviation or number and percentage in parentheses unless otherwise stated. All variables were tested for normal distribution using the Kolmogorov-Smirnov test. The Student's *t-*test was used to compare the means of continuous variables and normally distributed data. Otherwise, the Mann-Whitney *U* test was used for nonnormally distributed data. Categorical data were analyzed using the chi-square test. Finally, risk factors were assessed by univariate Cox regression analysis, and variables that were statistically significant (*P* < 0.05) were included in multivariate analysis by applying multiple Cox regression analysis based on forward elimination of data [11]. The cumulative survival curves as a function of time were generated using the Cox regression survival approach. All statistical tests were 2-tailed, with *P* values less than 0.05 being considered statistically significant. Data were analyzed using SPSS 12.0 software for Windows (SPSS, Inc., Chicago, IL).

## 3. Results

### 3.1. Subject Characteristics

The overall mortality rate was 38.5% (30/78) (Table 1). The mean age of the ESRD patients who underwent a bone marrow biopsy was 63.5 ± 17.2 years, and the patients were followed up for 19.3 ± 26.8 months. There were no significant differences in baseline variables between the survivors and nonsurvivors.

Unexplained anemia (44.9%) was the most common indication for bone marrow biopsy in both the survivors (47.9%) and nonsurvivors (40.0%) (Table 2). There were also no significant differences in the indications for a biopsy between the survivors and nonsurvivors. Furthermore, there were no significant differences in the laboratory variables between the survivors and nonsurvivors (Table 3).

### 3.2. Bone Marrow Biopsy Findings

The survivors had a higher incidence of abnormal megakaryocyte distribution (*P* = 0.001), band and segmented cells (*P* = 0.021), and lymphoid cells (*P* = 0.029) compared to the nonsurvivors (Table 4).

### 3.3. Causes of Mortality

The overall mortality rate was 38.5% (30/78) and the most common cause of mortality was sepsis (83.3%) followed by respiratory failure (10%) (Table 5).

### 3.4. Predictors of Mortality

**Figure 1:**
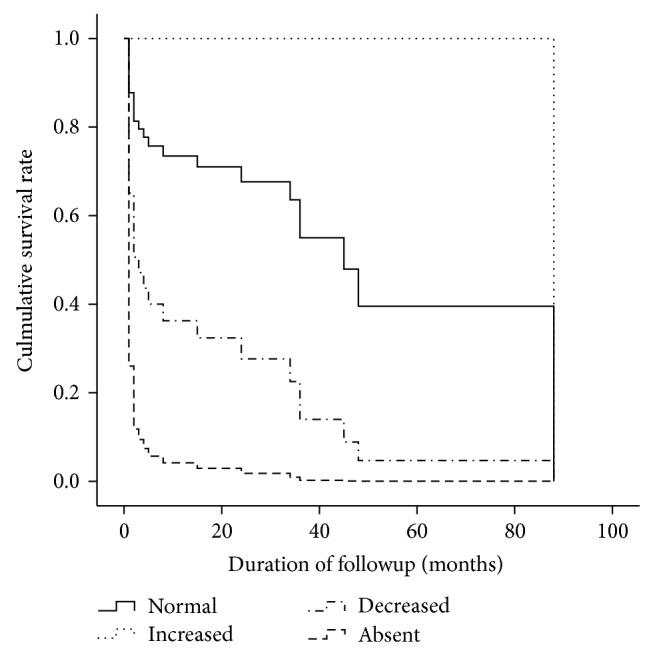
Kaplan-Meier survival probability estimates. The analysis showed a significantly higher cumulative mortality rate in the patients with a decreased and absent megakaryocyte distribution compared with those with a normal megakaryocyte distribution.

Univariate Cox regression analysis identified that systolic blood pressure, low bone marrow cellularity, megakaryocyte distribution, and M/E ratio were significantly associated with mortality (Table 6). In a multivariate Cox regression model, decreased (OR 3.714, 95% CI 1.671–8.253, *P* = 0.001) and absent (OR 9.751, 95% CI 2.030–45.115, *P* = 0.004) megakaryocyte distribution (normal megakaryocyte distribution as the reference group), as well as M/E ratio (OR 1.054, CI 1.012–1.098, *P* = 0.011), were predictive of mortality. The survival analysis showed a significantly higher cumulative rate of mortality in the patients with a decreased and absent megakaryocyte distribution compared with those with a normal megakaryocyte distribution (Figure 1).

## 4. Discussion

The M/E ratio is relevant to bone marrow function and also to diseases of bone marrow and peripheral blood such as leukemia and anemia. A normal M/E ratio is around 3 : 1, which may increase in patients with myelogenous leukemia and sepsis, decrease in patients with polycythemias, and reverse in cases of thalassemia [9]. In the current study, the M/E ratio of the nonsurvivors was 5.12, which is higher than that of the survivors (2.62; although not statistically significant) and higher than normal range. Furthermore, the most common cause of mortality in our patients was sepsis (83.3%) and the increase in M/E ratio may suggest that there was occult infection. Therefore, in a patient with high bone marrow M/E ratio, the possibility of occult infection should be taken into consideration.

The M/E ratio has also been reported to be higher in patients with erythroid hypoplasia, which can be caused by chronic inflammation [12], inadequate erythropoietin, or anti-erythropoietin antibodies in ESRD patients. Chan et al. [13] showed that growth of erythroid and granulocytic colonies was superior when cultured with nocturnal home hemodialysis (five to six times a week, 6 to 8 hours per session) plasma compared with conventional hemodialysis plasma and that intensification of the dosage of dialysis was associated with upregulation of the genes responsible for hematopoietic progenitor cell mobilization and growth and production of red blood cells. The conversion from conventional hemodialysis (three times a week, 4 hours per session) to nocturnal home hemodialysis has been reported to result in a three- to fourfold increase in urea clearance [14]. A direct relationship between the dosage of hemodialysis and the responsiveness of bone marrow in patients with ESRD has also been reported. Therefore, inadequate dialysis can be a cause of erythroid hypoplasia and result in anemia, which directly contributes to significant morbidity and mortality in ESRD patients [15].

The processes of megakaryocytopoiesis and platelet production occur within a complex bone marrow microenvironment where chemokines, cytokines, and adhesive interactions play a major role. Besides thrombopoietin, which is the main physiological regulator of megakaryocytopoiesis, other growth factors that stimulate megakaryocyte growth alone or in combination with EPO include granulocyte-macrophage colony-stimulating factor, interleukin-3 (IL-3), IL-6, IL-11, stem cell factor, Fms-like tyrosine kinase 3 ligand, fibroblast growth factor, and EPO [16, 17]. On the other hand, transforming growth factor-beta1, platelet factor 4, IL-4, and Src kinase inhibitors have been shown to negatively regulate megakaryocyte proliferation by inducing megakaryocyte differentiation and functional platelet-like fragment formation in vitro [18, 19]. Alvarez-Lara et al. showed that in uremic patients, only 5.1 ± 2.1% of the T lymphocytes contained interferon gamma (Th1 cells), while 61.9 ± 14.8% contained IL-4 (Th2 cells) (*P* < 0.0001) [20]. More IL-4 may be produced in patients receiving inadequate dialysis.

A decreased and absent megakaryocyte distribution were significant predictors of mortality in this study. There are many potential etiologies of decreased megakaryocytes such as cancer cells which infiltrate into the bone marrow and destroy megakaryocytes, aplastic anemia, toxic chemicals, radiation therapy or chemotherapy, genetic problems hindering the production of normal platelets, exposure to certain drugs or alcohol slowing the production of megakaryocytes, or simply viral infections. Nevertheless, the cause of a decreased or absent megakaryocytes in our dialysis patients remains unclear, and further research is warranted. Notably, Kantarjian et al. [21, 22] also showed that, in patients with chronic myelogenous leukemia, a decreased megakaryocyte distribution was a predictor of mortality and was associated with a poor prognosis. Therefore, the clinical significance of megakaryocyte findings in bone marrow warrants further research.

## 5. Conclusion

The results of a bone marrow biopsy can be used to assess the pathology, and, in addition, M/E ratio and abnormal megakaryocyte distribution can predict mortality in chronic hemodialysis patients.

## Figures and Tables

**Table 1:** Baseline data of the patients stratified according to survival status (*n* = 78).

Variable	Total (*n* = 78)	Survivors (*n* = 48)	Nonsurvivors (*n* = 30)	*P* value
Age (years)	63.5 ± 17.2	61.7 ± 18.06	66.4 ± 15.6	0.237
Female gender, *n* (%)	36 (46.2%)	21 (43.8%)	27 (56.3%)	0.645
Hypertension, *n* (%)	58 (74.4%)	38 (79.2%)	20 (66.7%)	0.288
Diabetes mellitus, *n* (%)	30 (38.5)	16 (33.3%)	14 (46.7%)	0.339
Smoking habit, *n* (%)	19 (24.4%)	12 (25.0%)	7 (23.3%)	1.000
Alcohol consumption, *n* (%)	11 (14.1%)	5 (10.4%)	6 (20.0%)	0.319
Antihypertensive agents, *n* (%)	58 (74.4%)	36 (75.0%)	22 (73.3%)	1.000
Lipid-lowering agents, *n* (%)	4 (5.1%)	3 (6.2%)	1 (3.3%)	1.000
Iron, *n* (%)	59 (75.6%)	38 (79.2%)	21 (70.0%)	0.421
Erythropoietin, *n* (%)	48 (61.5%)	32 (66.7%)	16 (53.3%)	0.339
Duration of followup (months)	19.3 ± 26.8	22.4 ± 30.0	14.3 ± 22.4	0.196
*Kt*/*V*	2.1 ± 0.8	2.09 ± 0.6	2.21 ± 0.4	0.120

**Table 2:** Indications for bone marrow biopsy stratified according to survival status (*n* = 78).

Variable	Total (*n* = 78)	Survivors (*n* = 48)	Nonsurvivors (*n* = 30)	*P* value
Unexplained anemia, *n* (%)	35 (44.9%)	23 (47.9%)	12 (40.0%)	0.021^*^
Unexplained leukocytosis and leukopenia, *n* (%)	11 (14.1%)	5 (10.4%)	6 (20.0%)	
Fever of unknown origin, *n* (%)	5 (5.1%)	2 (4.2%)	3 (10.0%)	
Suspected multiple myeloma, *n* (%)	11 (14.1%)	8 (16.7%)	3 (10.0%)	
Suspected lymphoma, *n* (%)	7 (9.0%)	3 (6.3%)	4 (13.3%)	
Pancytopenia, *n* (%)	15 (19.2%)	10 (20.8%)	5 (16.7%)	
Thrombocytosis, *n* (%)	1 (1.3%)	1 (2.1%)	0 (0.0%)	
Thrombocytopenia, *n* (%)	7 (17.9%)	5 (10.4%)	2 (6.7%)	
Suspected hemolytic-uremic syndrome, *n* (%)	1 (1.3 %)	1 (2.1%)	0 (0.0%)	

^*^ *P* < 0.05.

**Table 3:** Laboratory findings of the patients stratified according to survival status (*n* = 78).

Variable	Total (*n* = 78)	Survivors (*n* = 48)	Nonsurvivors (*n* = 30)	*P* value
Red blood cell (10^6^/uL)	2.93 ± 0.70	2.90 ± 0.70	2.97 ± 0.72	0.663
Hemoglobin (g/dL)	8.46 ± 1.83	8.41 ± 1.94	8.54 ± 1.67	0.757
Hematocrit (%)	25.53 ± 5.50	25.45 ± 5.80	25.66 ± 5.10	0.867
Mean corpuscular volume (fL)	88.22 ± 7.43	88.56 ± 6.85	87.67 ± 6.37	0.609
Mean corpuscular hemoglobin (pg/cell)	29.25 ± 2.48	29.24 ± 2.35	29.21 ± 2.90	0.954
Mean corpuscular hemoglobin concentration (g/dL)	33.20 ± 1.38	33.09 ± 1.25	33.38 ± 1.57	0.375
Red blood cell distribution width (%)	16.09 ± 3.03	16.65 ± 2.91	15.26 ± 3.06	0.051
Platelet (10^3^/uL)	167.07 ± 162.17	191.09 ± 182.44	129.43 ± 117.14	0.104
White blood cells (10^3^/uL)	8.21 ± 6.97	7.76 ± 5.75	8.89 ± 8.56	0.494
Blast cells (%)	2.00 ± 0.00	2.00 ± 0.00		
Myelocytes (%)	1.46 ± 1.11	1.13 ± 0.63	1.64 ± 1.31	0.484
Metamyelocytes (%)	2.39 ± 1.14	2.50 ± 0	2.36 ± 1.31	0.888
Band cells (%)	2.58 ± 2.32	2.73 ± 2.65	2.50 ± 2.23	0.831
Segmented cells (%)	67.71 ± 20.80	68.77 ± 19.16	66.16 ± 23.25	0.600
Eosinophils (%)	3.88 ± 5.00	4.60 ± 5.80	2.33 ± 1.86	0.123
Basophils (%)	0.94 ± 1.42	1.02 ± 1.62	0.78 ± 0.90	0.589
Monocytes (%)	7.43 ± 5.52	8.19 ± 5.77	6.28 ± 4.18	0.130
Lymphocytes (%)	19.01 ± 16.92	17.71 ± 15.06	20.88 ± 19.40	0.435
Atypical lymphocytes (%)	1.83 ± 1.34	1.70 ± 1.26	1.93 ± 1.48	0.786
Alanine aminotransferase (U/L)	19.16 ± 19.35	21.30 ± 22.36	15.94 ± 10.78	0.369
Blood urea nitrogen (mg/dL)	77.95 ± 51.71	74.46 ± 54.80	83.30 ± 47.05	0.486
Creatinine (mg/dL)	8.22 ± 4.60	8.66 ± 4.87	7.56 ± 4.15	0.320
Uric acid (mg/dL)	7.67 ± 3.93	7.61 ± 3.72	7.84 ± 4.92	0.915
Calcium (mg/dL)	8.80 ± 1.30	9.03 ± 1.28	8.45 ± 1.28	0.068
Phosphorus (mEq/L)	5.33 ± 2.56	5.04 ± 2.27	5.74 ± 2.91	0.261
Potassium (mEq/L)	4.34 ± 0.93	4.39 ± 0.96	4.26 ± 0.90	0.558
Bicarbonate (mEq/L)	20.65 ± 4.53	21.84 ± 4.32	18.96 ± 4.60	0.207
Serum iron (ug/dL)	35.20 ± 22.88	31.20 ± 20.16	36.13 ± 34.15	0.563
Total iron binding capacity (ug/dL)	202.40 ± 59.69	185.30 ± 47.53	245.27 ± 60.12	0.227
Ferritin (ng/mL)	837.90 ± 367.00	875.18 ± 312.12	749.85 ± 412.12	0.312

**Table 4:** Bone marrow biopsy findings stratified according to survival status (*n* = 78).

Variable	Total (*n* = 78)		Survivors (*n* = 48)	Nonsurvivors (*n* = 30)	*P* value
Cellularity, n (%)	Hypocellularity	40 (51.3)	20 (41.7)	20 (66.7)	0.077
	Normocellularity	23 (29.5)	18 (37.5)	5 (16.7)	
	Hypercellularity	15 (19.2)	10 (20.8)	5 (16.7)	

Megakaryocyte distribution, n (%)	Normal	57 (73.1)	39 (81.3)	18 (60.0)	0.001^**^
	Increased	6 (7.7)	6 (6.3)	0 (0)	
	Decreased	13 (16.7)	3 (6.3)	10 (33.3)	
	Absence	2 (2.6)	0 (0)	2 (6.7)	

Morphology, megakaryocytes, *n* (%)	Normal	77 (98.7)	48 (100.0)	29 (96.7)	0.385
	Dysplasia	1 (1.3)	0 (0)	1 (3.3)	

M/E ratio	3.5 ± 5.98		2.62 ± 1.64	5.12 ± 9.30	0.154

Myeloid series (%)	50.73 ± 17.28		53.06 ± 16.21	47.08 ± 18.53	0.140

Blast cells (%)	0.90 ± 1.06		1.02 ± 1.19	0.70 ± 0.77	0.213

Promyelocytes (%)	2.38 ± 3.16		2.47 ± 3.71	2.22 ± 1.87	0.743

Myelocytes + metamyelocytes (%)	18.52 ± 9.81		18.08 ± 9.29	19.23 ± 10.70	0.618

Band cell + segmental cell (%)	29.58 ± 14.16		32.48 ± 14.38	24.94 ± 12.70	0.021^*^

Morphology, myeloid series	Normal	77 (98.7)	48 (100.0)	29 (96.7)	0.194
	Abnormal	1 (1.3)	0 (0)	1 (3.3)	

Erythroid series (%)	23.99 ± 12.49		24.98 ± 11.11	22.39 ± 14.48	0.407

Morphology, erythroid series	Normal	77 (98.7)	48 (100.0)	29 (96.7)	0.328
	Dysplasia	1 (1.3)	0 (0)	1 (3.3)	

Monohistiocytes (%)	1.07 ± 1.23		1.10 ± 1.32	1.02 ± 1.14	0.814

Eosinophils (%)	3.41 ± 3.23		3,91 ± 3.19	2.60 ± 3.19	0.085

Plasma cells (%)	4.40 ± 10.25		2.64 ± 2.24	7.35 ± 16.28	0.139

Lymphoid cells (%)	15.59 ± 11.56		12.99 ± 7.77	19.74 ± 15.10	0.029^*^

Iron store (grade)	2.41 ± 1.33		2.35 ± 1.16	2.54 ± 1.66	0.713

^*^ *P* < 0.05, ^**^ *P* < 0.01.

**Table 5:** Causes of mortality (*n* = 30).

Variable	
Sepsis, *n* (%)	25 (83.3)
Respiratory failure, *n* (%)	3 (10.0)
Liver failure with encephalopathy, *n* (%)	1 (3.3)
Intracerebral hemorrhage	1 (3.3)

**Table 6:** Analysis of risk factors for mortality using Cox regression analysis (*n* = 78).

	*B*	SE	Exp(*B*)	*P* value
*Univariate *				
Systolic blood pressure (mmHg)	−0.019	0.009	0.982 (0.963–1.000)	0.049^*^
Low bone marrow cellularity	−0.021	0.01	0.979 (0.960–0.999)	0.042^*^
Megakaryocyte distribution^#^				
Increased	−13.309	656.54	0.000	0.984
Decreased	1.312	0.407	3.714 (1.671–8.253)	0.001^**^
Absent	2.259	0.791	9.571 (2.030–45.115)	0.004^**^
M/E ratio	0.047	0.018	1.048 (1.012–1.087)	0.009^**^
Plasma cell number	0.026	0.011	1.027 (1.005–1.049)	0.014^*^
Lymphoid cell number	0.036	0.013	1.037 (1.010–1.064)	0.006^**^
*Multivariate *				
Megakaryocyte distribution^#^				
Increased	−13.419	636.058	0.000	0.983
Decreased	1.192	0.415	3.292 (1.461–7.420)	0.004^**^
Absent	2.335	0.808	10.333 (2.119–50.376)	0.004^*^
M/E ratio	0.053	0.021	1.054 (1.012–1.098)	0.011^*^

^#^Normal megakaryocyte distribution as reference, ^*^ *P* < 0.05, ^**^ *P* < 0.01.
